# Knowledge, Attitudes, Practices, and Acceptability of Medical Male Circumcision among Males in Traditionally Circumcising Rural Communities of Alfred Nzo District, Eastern Cape, South Africa

**DOI:** 10.3390/ijerph20237091

**Published:** 2023-11-21

**Authors:** Thobani Ntshiqa, Alfred Musekiwa, Riyadh Manesen, Hetani Mdose, Nqobile Ngoma, Lazarus Kuonza, Thomas Dlamini, Carl Reddy, Seymour Williams

**Affiliations:** 1 South African Field Epidemiology Training Programme, National Institute for Communicable Disease, Sandringham, Johannesburg 2131, South Africa; alfred.musekiwa@up.ac.za (A.M.); hetanim@nicd.ac.za (H.M.); nqobilen@nicd.ac.za (N.N.); lazarusk@nicd.ac.za (L.K.); creddy@tephinet.org (C.R.); sjw9@cdc.gov (S.W.); 2 South African National Aids Council, Pretoria 0002, South Africa; 3 School of Health Systems and Public Health, University of Pretoria, Pretoria 0002, South Africa; 4 The Aurum Institute, Johannesburg 2193, South Africa; r.manesen@think.org.za; 5 GERMS-SA, National Institute for Communicable Disease, Sandringham, Johannesburg 2131, South Africa; 6 Epidemiology Unit, Eastern Cape Department of Health, Bisho 5605, South Africa; thomas.dlamini@echealth.gov.za; 7 TEPHINET Secretariat, The Task Force for Global Health, Decatur, GA 30030, USA; 8 Centers for Disease Control and Prevention, Atlanta, GA 30328, USA

**Keywords:** medical male circumcision, knowledge, attitude, practices, acceptability, traditional circumcising communities, HIV prevention, South Africa

## Abstract

Male circumcision (MC) reduces HIV transmission risk by up to 60% in heterosexual men. However, uptake of medical male circumcision (MMC) is low in traditionally circumcising communities of South Africa. We assessed knowledge, attitudes, and practices to identify factors predicting acceptability of MMC among males in the Alfred Nzo District. A cross-sectional study was conducted among males aged 15–49 years in this district. Logistic regression was used to identify factors predicting acceptability of MMC. We interviewed 343 males who had a median age of 19 years (interquartile range (IQR): 16–25 years). Of these, 77% (95% confidence interval (CI): 72–82) were circumcised: 77% (95% CI: 71–82) were circumcised in a traditional setting and 21% (95% CI: 16–26) in a medical setting. The median score of knowledge about the benefits of MMC was 62.5% (IQR: 37.5–75.0), with 59% (95% CI: 53–64) demonstrating a positive attitude towards MMC and 68% (95% CI: 63–73) accepting involvement of health workers in MC. Excellent knowledge (adjusted odds ratio (aOR): 3.07, 95% CI: 0.99–9.58, *p* = 0.053), awareness (aOR: 3.26, 95% CI: 1.08–9.86, *p* = 0.037), and positive attitude towards MMC (aOR: 2.35, 95% CI: 1.30–4.25, *p* = 0.005) were associated with acceptability of MMC. Participants demonstrated good knowledge and acceptance of the MMC programme. Knowledge, attitude, and awareness were significant predictors of MMC acceptability.

## 1. Introduction

Male circumcision (MC) is a well-established practice involving surgical removal of the foreskin of the penis and is practiced in different parts of the world for medical, religious, and cultural reasons [1,2,3,4]. It is commonly conducted in clinical and traditional settings among males during infancy, childhood, adolescence, or adulthood [2]. Globally, religion is the major determinant of MC and in some communities such as Muslim and Jewish communities, MC is a universal practice [2]. In Sub-Saharan Africa, stark regional contrast had been observed with central and western Sub-Saharan African regions reporting higher MC practices compared to southern Sub-Saharan Africa [5].

In South Africa, MC is highly practiced with culture being a major driver, usually in the form of traditional circumcision. This is predominantly by certain ethnic groups including AmaXhosa (Eastern Cape Province), AmaVhendha, Southern Sotho (Limpopo Province), and AmaNdebele (Mpumalanga Province) [6,7,8]. Traditional male circumcision (TMC) is a very secretive ritual performed in the “mountains” by traditionally trained male surgeons, a person performing MC who often has no medical background or medical training, using traditionally available instruments and techniques, and typically without the use of any anaesthetic or analgesic [2,9]. The ritual is performed as a rite of passage for young adult males with the main purpose of integrating the male child into adult society, building his character, shaping personal discipline, transferring livelihood and family life skills, and building community life and village systems [2,9,10].

Since 1986, more than 40 studies observed a strong correlation between MC and HIV prevalence globally [8,10]. However, the evidence presented by these studies was based on observational data; therefore, the causal link between MC and HIV incidence could not be determined. Between 2005 and 2006, three randomized controlled trial (RCT) studies were conducted in three Sub-Saharan African countries to explore the potential link between MC and HIV infection. These studies found that the incidence of HIV was significantly lower (76%, South Africa; 60%, Kenya; and 55%, Uganda) among circumcised men compared to uncircumcised men [1,3,4]. A meta-analysis of these RCT studies with more than 40 observational studies suggested a 60% protective effect of female-to-male HIV transmission [11].

Plausible biological explanations for this protective effect include removal of HIV target cells (Langerhan’s cells and CD4 T-cells) found in the foreskin and keratinization [12]. The removal of the foreskin also promotes the keratinization (hardening) of the skin surface [12]. Keratinization reduces the likelihood of bacterial sexual infections (chancroid) and the risk of HIV acquisition [13]. Furthermore, the soft mucosal surface of the inner foreskin is highly receptive to HIV compared to outer foreskin tissue [13]. Inner foreskin tissue readily absorbs HIV up to nine times more easily than cervical mucosal tissue [13].

The compelling scientific evidence from these studies is widely recognized and has influenced the HIV policy position at global and regional levels. As a result, in March 2007, medical male circumcision (MMC) was recommended by the World Health Organization (WHO) and Joint United Nations Programme on HIV/AIDS (UNAIDS) as a biomedical intervention tool [14]. Voluntary male medical circumcision (VMMC) is now an intervention programme for HIV prevention in most Sub-Saharan African countries [14].

In 2010, the South African government introduced the VMMC as part of its comprehensive HIV prevention strategy, with the primary objective of ensuring a multi-pronged approach to HIV prevention [7,15]. Mathematical modelling estimated that circumcising 80% of males aged 15 to 49 years could avert more than one million HIV infections within a 5-year period [16]. The ability to meet this goal was limited by two challenges, namely acceptability and client demand.

In 2012, the prevalence of HIV was 20% among the adult population in Eastern Cape [7]. During the same period, the prevalence of MC was 74%, with TMC accounting for 83% and MMC accounting for only 14% of the reported circumcisions [7]. Alfred Nzo is a rural district in the Eastern Cape Province, made up of the Maluti and Umzimvubu sub-districts. The majority of the population (99.1%) in Alfred Nzo is Black with Xhosa ethnic origins (84.6%) [17]. TMC is an integral part of the culture and customs of the communities in this district. Most often, TMC involves partial removal of the foreskin and sharing of the “knife”, posing an increased risk for HIV transmission. In 2012, the prevalence of HIV was 16% to 22% among the adult population in Alfred Nzo [7]. Within traditional circumcising communities such as Alfred Nzo District, demand and acceptability of MMC is lower due to many factors including stigma and discrimination associated with being medically circumcised [18,19,20].

Observational studies have shown that circumcisions undertaken in non-clinical settings have an increased risk of men developing serious post-operative complications, including death [21,22,23]. Accounts of serious complications and adverse events following circumcision in traditional settings in the Eastern Cape Province have been widely reported in the past [21,22,23]. A systematic review has shown a strong correlation between MC and health benefits including safety and reduced risk in acquisition of HIV and urinary tract infections, syphilis, chancroid, and the human papilloma virus [24]. Furthermore, in female sexual partners of those that underwent MC, it reduces the risks of developing cervical cancer by 2 to 6 times [25,26].

Understanding and addressing barriers of MMC is an urgent priority to inform effective demand-creation strategies. Targeted efforts are therefore needed to understand what factors influence the demand and low uptake of MMC in traditional circumcising communities such as Alfred Nzo District. Furthermore, scaling up MMC as an HIV prevention strategy could significantly reduce and mitigate the HIV incidence in traditional circumcising communities. This study described knowledge, attitudes, practices, perceptions, and acceptability of MMC in the Alfred Nzo District. The study also assessed factors predicting knowledge, attitude, practices, and acceptability of MMC among males aged 15 to 49 years in Alfred Nzo District in 2015.

## 2. Materials and Methods

### 2.1. Study Design

This study was a cross-sectional study with descriptive and analytical components, which was conducted from 27 June 2015 to 21 August 2015. The eligible participants were South African Black males aged 15 to 49 years residing in Alfred Nzo District. The study was conducted in the rural communities of Alfred Nzo District in the Eastern Cape Province, South Africa. Alfred Nzo District is a rural district, which is estimated to be about 6858 km^2^ comprising of two sub-districts, i.e., Maluti (2352 km^2^) and Umzimvubu (4506 km^2^). The total population is estimated to be about 395,466. Participants were included in the study only if they were willing and consented to be part of the study. Participants under the age of 18 years were requested to sign an assent form for inclusion in the study. Both circumcised and uncircumcised males participated in the study. Women and non-Indigenous males were excluded.

A multi-stage stratified random sampling was used to select study participants. First, we stratified Alfred Nzo District into its two sub-districts: Maluti and Umzimvubu. Then, we stratified each sub-district into its rural communities. In South Africa, rural communities are defined as “areas that include large settlements in the former homelands, which depend on migratory labour and remittances as well as government social grants for their survival, and typically have traditional land tenure systems” [17]. From each rural community, we selected areas containing clusters of households using simple random sampling. Then, households within each selected area were selected using simple random sampling. Finally, two males per household were asked to participate in the study. Participants were also recruited in community meetings and high schools in the absence or lack of community participation.

Since the prevalence of knowledge, attitudes, practices, and acceptability of MMC in Eastern Cape Province is not known, we assumed a prevalence of 50%, which, mathematically, would give us the maximum sample size for a cross-sectional survey. With 50% prevalence for a cross-sectional study, a population of 395,466, a 95% confidence level width of 5%, and 5% level significance, a minimum sample size of n = 385 was obtained using Epi-Info 7 statistical software. To allow a 90% response rate, and effect design of ≤0.1, the final sample size required for this study was *n* = 467.

### 2.2. Measurements, Quality Control, and Pilot Study

A structured questionnaire tool was printed and used to collect data. The questionnaire tool was structured into five sections. Section A captured demographic characteristics; section B captured knowledge; section C included attitude, perceptions, and acceptability data; and section D consisted of circumcision status and practices information. Section E captured data among male parents. To ensure internal validity, the questionnaire tool was translated from English to IsiXhosa. The translated version was also translated back to English. Furthermore, the questionnaire was pilot-tested to address ambiguities prior to the data collection process.

The data collection process was closely monitored throughout the study to prevent potential errors. Before and during the survey, a range of quality control measures were put in place to ensure that collection and recording were based on high data quality and acceptable scientific standards. Four data collectors were trained to collect data in a standardized manner and all interviews were conducted in IsiXhosa from a structured questionnaire, which was interviewer-administered.

Internal validity of the tool was also measured using the Cronbach alpha coefficient. A Cronbach alpha coefficient of 0.70 or above was considered adequate to show internal consistency and reliability of the questionnaire. Questionnaire items were therefore revised until they had an acceptable Cronbach alpha coefficient. Fifteen male participants who met the inclusion criteria were randomly selected for the pilot study. During the piloting process, we tested the data collection tool to assess if there were any ambiguities before administering the tool to all study participants. Where ambiguities were found, the data-collection instrument was modified before we started the enrolment process.

### 2.3. Data Analysis

Frequency distribution tables, proportions, and a bivariate analysis were used to describe knowledge, practices, and acceptability of MMC. Chi–square testing was used to assess the association between categorical variables and infer statistical significance. Logistic regression was used to assess predictors of variables with binary outcomes, and an ordered logistic regression model was used to determine predictors of excellent knowledge. On the bivariate model, a cut-off *p*-value of ≤0.15 in the logistic regression was used to maintain variables, which were introduced into the bivariate and multivariate models. A manual forward selection stepwise procedure was used to obtain unadjusted and adjusted odds ratios, 95% confidence intervals (CIs), and p-values. Ordered logistic regression was used to determine predictors of excellent knowledge. All statistical analyses were performed using Stata version 15 (StataCorp. 2015. Stata Statistical Software: Release 15. College Station, TX, USA: StataCorp LP). A *p*-value of <0.05 was considered statistically significant.

### 2.4. Variables

#### 2.4.1. Knowledge

The questionnaire included eight knowledge questions. Participants who answered all the knowledge questions correctly were assigned a total score of 8. A knowledge score of 2 or less was considered poor; a knowledge score above 2 to 4 was considered fair knowledge; above 4 to 6 was considered good; and a knowledge score above 6 was considered excellent knowledge.

#### 2.4.2. Attitudes

A score of 3 was given to any individual who responded positively to all questions; 2 for two “positive” answers; 1 for one “positive” answer; and 0 for all “negative” answers. These scores were then dichotomized into “0” (we combined 0 and 1) and “1” (we combined 2 and 3). The “0” response indicated a negative attitude and “1” indicated a positive attitude.

#### 2.4.3. Practices

The questionnaire collected data on circumcision status among all study participants. If circumcised, participants were asked (i) who conducted the circumcision (traditional surgeon vs. medical personnel) and (ii) where was the circumcision conducted (clinical vs. non-clinical settings).

#### 2.4.4. Perceptions and Beliefs

A four-point Likert scale was used to collect data on perceptions and beliefs of study participants towards MC. Statements were generated from existing literature on common perceptions and beliefs towards MC among men. All participants were asked to indicate the extent to which they agreed with the statements regarding the practice of MC. During the analysis, responses were binarized into two outcomes, “Agree” and “Do not agree”. Proportions were used to summarize the variables and compare those who agree versus those who did not agree with the statements.

#### 2.4.5. Acceptability

A question with binary outcomes “Yes” or “No” was asked of all participants to determine if they accepted or would accept the involvement of health workers in MC. Uncircumcised participants were asked if they would consider being medically circumcised in the future, while male parents were asked if they would support MMC for their sons.

### 2.5. Ethical Considerations

The study received ethical clearance from University of Pretoria Faculty of Health Sciences Research Ethics Committee (reference number: 440/2014), and Eastern Cape Provincial Department of Health Research Ethics Committee (reference number: EC_2015RP42_600). All persons who participated in this study signed a written consent and participation was voluntary. Privacy and confidentiality were maintained throughout the interviews and questionnaires were filled anonymously.

## 3. Results

### 3.1. Data Collection

From 1st to 26th of June 2015, the study team conducted stakeholder engagements from provincial to village levels before the commencement of enrolment activities to seek permission and sensitize communities. Following these extensive stakeholder engagements, the study team approached the selected households from 27 June to 21 August 2015. A total of 424 potential participants were approached in households and high schools and were invited to participate in the study. Of those who were approached, 92% initially paid attention to study staff, and 83% agreed to participate and signed consent and/or assent. Of those who provided consent and/or assent, 2% of records were excluded from the analysis due to incomplete data (completeness <80%). Therefore, 81% were eligible for this analysis (data completeness ≥80%).

### 3.2. Demographic Characteristics of Study Participants

We enrolled 343 male participants in this study whose median age was 19 years (IQR: 16–25). Of these, 59% were aged <20 years old and 41% were aged ≥20 years. Most participants were Xhosa speaking (81%) and Black males (91%), with 88% having obtained secondary education. Forty-one percent of respondents originated from Mbizana, which is the most populous rural town within Alfred Nzo District. AmaXhosa (43%) and AmaMpondo (29%) were the most represented tribes. Christianity (54%) was the most dominant religion, while 41% reported Traditional African Religion (traditionalists) as their religion (Table 1).

### 3.3. Knowledge about the Benefits of Medical Male Circumcision

Seventy one percent (95% CI: 65–75) of the study participants knew that MC reduces chances of HIV infection and believed MC does not completely protect against HIV infection. Furthermore, 69% (95% CI: 64–74) knew that circumcised men can be infected with HIV if a condom is not used during sexual intercourse. Additionally, 52% (95% CI: 47–58) believed that circumcision of a HIV-positive man does not protect his sexual partner from being infected with HIV. However, 38% (95% CI: 32–43) knew that circumcision reduces the chance of becoming infected with sexually transmitted infections. Additionally, 57% (95% CI: 51–62) and 44% (95% CI: 38–49) of the study participants perceived being uncircumcised as a greater risk for HIV infection and sexually transmitted infections, respectively. However, 25% (95% CI: 20–30) believed that there was no association between being circumcised and HIV infection while 11% (95% CI: 7–14) had no knowledge about the association between HIV infection and circumcision.

#### Level of Knowledge about the Benefits of Medical Male Circumcision

The median knowledge about the benefits of MMC was 62.5% (IQR: 37.5–75.0). Only 16% (95% CI: 12–20) of the study participants demonstrated poor knowledge, while 33% (95% CI: 28–38) demonstrated a fair knowledge about the benefits of MMC. Moreover, 31% (95% CI: 25–35) had good knowledge about the benefits of MMC, with the remaining 20% (95% CI: 16–25) demonstrating excellent knowledge about the benefits of MMC.

On the bivariate analysis, poor knowledge of the benefits of MMC was associated with being aged <20 years (20%, 95% CI: 14–26, *p* = 0.021); being a traditionalist compared to being Christian (21%, 95% CI: 15–29, *p* = 0.009); being unemployed (11%, 95% CI: 5–22, *p* = 0.037); having a negative attitude towards MMC (22%, 95% CI: 15–30, *p* < 0.001); being uncircumcised (32%, 95% CI: 22–44, *p* < 0.001); being traditionally circumcised (12%, 95% CI: 8–17, *p* < 0.001); and being from Mbizana (18%, 95% CI: 12–25, *p* = 0.001) (Table 2).

Excellent knowledge of the benefits of MMC was associated with being aged ≥20 years (27%, 95% CI: 19–35, *p* = 0.021); being a Christian (27%, 95% CI: 22–37, *p* = 0.009); being unemployed (33%, 95% CI: 21–46, *p* = 0.037); having a positive attitude towards MMC (27%, 95% CI: 21–34, *p* < 0.001), being circumcised (22%, 95% CI: 17–28, *p* < 0.001); being medically circumcised (49%, 95% CI: 35–63, *p* < 0.001); and being from Ntabankulu (40%, 95% CI: 28–53, *p* = 0.001) (Table 2).

On the ordered logistic regression model, having a positive attitude towards MMC (OR: 1.80, 95% CI: 1.09–2.98, *p* = 0.023) and being medically circumcised (OR: 2.62, 95% CI: 1.38–4.99, *p* = 0.003) were found to be significantly associated with having excellent knowledge about the benefits of MMC.

### 3.4. Attitudes towards Medical Male Circumcision

Sixty eight percent (95% CI: 63–73) of study participants accepted the involvement of healthcare workers in MC. Although 42% (95% CI: 37–48) of the study participants indicated that they would not be friends with medically circumcised men, 59% (95% CI: 53–64) demonstrated a positive attitude towards MMC (Table A1).

#### Predictors of Positive Attitude towards Medical Male Circumcision

On the multivariate analysis, being aged ≥20 years (aOR: 2.65, 95% CI: 1.29–5.45, *p* = 0.008); being from Ntabankulu (aOR: 4.36, 95% CI: 1.25–15.18, *p* = 0.021); being Hlubi (aOR: 5.15, 95% CI: 1.01–25.94, *p* = 0.047); being Mpondo (aOR: 4.05, 95% CI: 1.06–15.47, *p* = 0.041); being Xhosa (aOR: 3.58, 95% CI: 1.04–12.37, *p* = 0.044); being a Christian (aOR: 2.65, 95% CI: 1.40–5.03, *p* = 0.003); being medically circumcised (aOR: 16.08, 95% CI: 4.19–61.75, *p* < 0.001); and being uncircumcised (aOR: 2.65, 95% CI: 1.40–5.03, *p* = 0.003) remained independently associated with having a positive attitude towards MMC (Table 3).

### 3.5. Perceptions and Beliefs about Male Circumcision

Eighty five percent 80% (95% CI: 80–88) of the study participants believed that circumcised men enjoy sex more than uncircumcised men. Males aged ≥20 years (92%, 95% CI: 86–96, *p* = 0.006); those who were employed (93%, 95% CI: 81–99, *p* = 0.036); or those circumcised (91%, 95% CI: 87–94, *p* < 0.001) were more likely to believe this.

The majority (70%, 95% CI: 65–75) of participants believed that circumcision proves manhood with most (73%, 95% CI: 68–78) saying traditional circumcision is an old traditional practice and therefore does not need to be changed or re-introduced. Older men (74%, 95% CI: 66–81, *p* < 0.001); those who were AmaHlubi (77%, 95% CI: 56–91, *p* = 0.019); those who were unemployed (79%, 95% CI: 65–87, *p* = 0.001); those from Ntabankulu (86%, 95% CI: 75–93, *p* = 0.012); those from Maluti (73%, 95% CI: 54–87); or those circumcised (77%, 95% CI: 71–82, *p* < 0.001) were more likely to believe that circumcision proves manhood.

Fifty seven percent (95% CI: 51–62) of respondents believed that all men, irrespective of circumcision status, still needed to use a condom to prevent HIV infection. However, males aged ≥20 years (36%, 95% CI: 28–45, *p* < 0.001) or who were AmaXhosa (37%, 95% CI: 29–45, *p* = 0.005); traditionalist (34%, 95% CI: 26–42, *p* = 0.001); unemployed (43%, 95% CI: 30–56, *p* = 0.035); from Ntabankulu (44%, 95% CI: 32–57, *p* < 0.001); or circumcised (34%, 95% CI: 29–40, *p* = 0.037) were more likely to believe that circumcised men can safely have sex without using a condom without becoming infected with HIV.

In total, 82% (95% CI: 77–87) of those who were circumcised believed that MC is protective against HIV infection; this included 80% (95% CI: 74–85) who were traditionally circumcised and 91% (95% CI: 80–97) who were medically circumcised. When asked about the level of protection provided by circumcision, 26% (95% CI: 21–32) did not know the level of protection; 9% (95% CI: 6–13) believed it was less than 50%; and 20% (95% CI: 15–25) indicated it was 60–69%.

Twenty nine percent (95% CI: 24–35) of circumcised men believed that circumcision is 100% protective against HIV infection. Those aged <20 years (27%, 95% CI: 21–34, *p* = 0.043) or who were single (22%, 95% CI: 17–28, *p* = 0.054); had secondary education (24%, 95% CI: 19–29, *p* = 0.033); students (29%, 95% CI: 23–35, *p* < 0.001), from Mount Frere (36%, 95% CI: 23–50, *p* < 0.001); or traditionally circumcised (32%, 95% CI: 26–39, *p* < 0.001) were more likely to believe that MC is 100% protective against HIV infection.

### 3.6. Circumcision Practices

The prevalence of MC was 77% (95% CI: 72–82) in the Alfred Nzo District. Of these, 21% (95% CI: 16–27) were medically circumcised and 79% (95% CI: 73–84) were traditionally circumcised (Table A1). Most men were either circumcised during adolescence (13–17 years) (52%, 95% CI: 46–58) or at adulthood (18 years or above) (42%, 95% CI: 36–48). Ninety six percent (95% CI: 91–98) of those aged ≥20 years indicated that they were circumcised, suggesting that there were few (four percent, 95% CI: 2–9) circumcisions performed above the age of 20 years.

The majority (60%, 95% CI: 55–65) of men indicated that culture was the main reason why they wanted to be circumcised. Other reasons cited as motivators for MC included HIV prevention (18%, 95% CI: 14–22), medical benefits (13%, 95% CI: 9–17), religion (10%, 95% CI: 7–14), hygiene (10%, 95% CI: 7–14), and sexual pleasure (7%, 95% CI: 5–11). Most (80%, 95% CI: 75–85) circumcised men indicated that they were circumcised in the traditional setting and 77% (95% CI: 71–82) of them were circumcised for cultural reasons. Similarly, 64% (95% CI: 52–75) of uncircumcised men were also motivated by culture to be circumcised in the future. However, culture (51%, 95% CI: 46–56) and fear of pain (17%, 95% CI: 13–21) were found to be major barriers for MMC in the Alfred Nzo District.

Nineteen percent (95% CI: 14–25) of traditionally circumcised men reported that they never used a condom after circumcision, while only six percent (95% CI: 1–15) of medically circumcised men reported this. Half (50%, 95% CI: 43–57) of traditionally circumcised men reported inconsistent condom use, while only 36% (95% CI: 23–49) of medically circumcised men reported this. Medically circumcised men were more likely to seek HIV testing about every 3 months compared to traditionally circumcised men.

Sixty five percent (95% CI: 59–71) of circumcised men indicated that they were not aware of their HIV statuses and traditionally circumcised men (seventy percent, 95% CI: 63–76, *p* = 0.001) were more likely to be unaware of their HIV status. Thirty eight percent (95% CI: 29–48) of male parents indicated that their sons were circumcised. Of these, 58% (95% CI: 42–73) were traditionally circumcised while 42% (95% CI: 27–58) were medically circumcised.

Thirty eight percent (95% CI: 32–43) of men indicated that they would not have friendships with uncircumcised men and 42% (95% CI: 37–48) indicated that they would not have friendships with medically circumcised men. Culture (39%, 95% CI: 34–44) and social norms (43%, 95% CI: 38–49) were major reasons for rejecting uncircumcised and medically circumcised men.

#### Predictors of Being Medically Circumcised

On the multivariate analysis, being aged ≥20 years (aOR: 2.44, 95% CI: 1.23–4.84, *p* = 0.011); having excellent knowledge about the benefits of MMC (aOR: 3.38, 95% CI: 1.09–11.49, *p* = 0.050); and being aware of the association between circumcision and HIV infection (aOR: 3.26, 95% CI: 1.08–9.86, *p* = 0.037) remained significantly associated with the practice of MMC (Table 4).

### 3.7. Acceptability of Medical Male Circumcision

Sixty eight percent (95% CI: 63–73) of the study participants accepted the involvement of healthcare workers in MC. Of this, 76% (95% CI: 70–82) were circumcised and 24% (95% CI: 18–30) were uncircumcised. Of the 78 uncircumcised males, 71% (95% CI: 59–80) accepted healthcare worker involvement. Of the 265 circumcised males, 67% (95% CI: 61–73) accepted the involvement of the healthcare workers in MC. Forty nine percent (95% CI: 37–60) of the uncircumcised men indicated that they would consider being medically circumcised and sixty nine percent (95% CI: 59–77) of male parents supported MMC for their sons.

Fifty five percent (95% CI: 49–60) of our study participants reported that the ideal age for MC is 18 years or above. However, some (31%, 95% CI: 26–36) believed 17 years or below was the ideal age for circumcision. Most parents also preferred MC to be conducted during adulthood (61%, 95% CI: 51–70) rather than childhood or adolescence (39%, 95% CI: 30–49). When asked who they preferred to conduct the circumcision procedure, 47% (95% CI: 42–52) preferred a traditional circumciser, 38% (95% CI: 33–44) preferred a medical doctor, and 6% (95% CI: 3–9) preferred a nurse, while 9% (95% CI: 6–13) had no opinion. The majority (64%, 95% CI: 59–69) of study participants believed that MC, irrespective of circumcision type, should be conducted in a traditional setting rather than at a health facility (25%, 95% CI: 20–30), home (2%, 95% CI: 1–4), or religious setting (1%, 95% CI: 0.3–3).

Although the majority (68%, 95% CI: 63–73) of study participants accepted the involvement of healthcare workers in MC, most indicated that this involvement should be limited to a certain degree. For instance, 50% (95% CI: 45–56) indicated that healthcare workers should only screen initiates before MC is conducted; 29% (95% CI: 25–35) indicated that male healthcare workers should conduct MC provided that they themselves are circumcised; 24% (95% CI: 19–28) believed that healthcare workers should be involved in wound care only when there are complications; and 6% (95% CI: 4–9) wanted healthcare workers to take over the circumcision practice. Thirty seven percent (95% CI: 32–42) of respondents indicated that they would choose to be medically circumcised if it reduces the risk of HIV infection. On the other hand, 39% (95% CI: 34–44) indicated that they would consider MMC if it was associated with less or no complications. Forty three percent (95% CI: 38–48) of respondents indicated that they would recommend MMC for a son or any young male (Table A1).

#### Factors Associated with Acceptability of Medical Male Circumcision

On the multivariate analysis, being Mpondo or Xhosa (aOR: 5.05, 95% CI: 1.53–16.66, *p* = 0.008); having excellent knowledge about the benefits of MMC (aOR: 3.07, 95% CI: 0.99–9.58, *p* = 0.053); having a positive attitude towards MMC (aOR: 2.35, 95% CI: 1.30–4.25, *p* = 0.005); and being medically circumcised (aOR: 2.81, 95% CI: 1.17–6.74, *p* = 0.021) remained significantly associated with the acceptability of MMC as a biomedical intervention (Table 5).

## 4. Discussion

We described the practices, knowledge, attitudes, beliefs, and acceptability of MMC among males in the Alfred Nzo District. We also determined factors predicting the level of knowledge, positive attitude, practice, and acceptability of MMC.

We found that 77% of males in Alfred Nzo District were circumcised. Of these circumcised males, 79% were circumcised in a traditional setting and 21% in a medical setting. The prevalence of MC reported in this study was slightly different from the average circumcision prevalence reported in 2012 in the Eastern Cape Province [7,27]. The Human Science Research Council found that about 74% of males in the Eastern Cape Province were circumcised in 2012; of these, 83% were traditionally circumcised while 14% were medically circumcised [7]. However, the third National HIV Communication Survey found, in the same year, a slightly lower average of 64%, where 77% were traditionally circumcised and 23% medically circumcised [27]. Although there is no significant difference between these findings, the increase and decline in the practice of MMC and TMC, respectively, may be indicative of a shift from the practice of TMC to MMC. This observation could be further supported by a high (68%) number of participants from this study who accepted the involvement of healthcare workers in MC.

Although most participants accepted the involvement of healthcare workers in MC, traditional circumcisers (50%) were still preferred over healthcare workers (48%). The majority (64%) of study participants believed that MC, irrespective of circumcision type, should be conducted in a traditional setting rather than at a health facility. As a result, most (80%) circumcised men were circumcised in the traditional setting, which is similar to findings reported in a systematic review conducted in southern Africa [28]. The preference of a traditional setting over a medical setting and traditional circumciser over healthcare workers could be explained by cultural sensitivity and men’s attitude against the involvement of nurses (predominantly women) in the medical setting. Despite this, our findings suggest that circumcising communities are more likely to accept the integration of MMC with TMC if circumcised male healthcare workers could conduct MMC in the traditional setting.

One of the existing benefits of TMC includes integration of a male child into adult society, building his character, shaping personal discipline, transferring livelihood and family life skills, and building community life and village systems [2,9,10]. The disadvantages include increased risk of developing serious post-operative complications, including death [21,22,23]. Accounts of serious complications and adverse events following circumcision in traditional settings in the Eastern Cape Province have been widely reported in the past [21,22,23]. On the other hand, MMC is safer and its integration to TMC may significantly reduce complications associated with TMC in traditionally circumcising communities.

The reluctance of males to access circumcision services at local health facilities might be indicative of a need for transformation in the healthcare system to respond appropriately to the cultural needs of people at the community level. Although HIV prevention, safety, and medical benefits were cited as reasons for circumcision, the majority of circumcised men (60%) and uncircumcised men (64%) were motivated by culture to perform circumcision. Interestingly, culture (51%) and fear of pain (17%) were found to be major barriers of MMC in the Alfred Nzo District. Fear of pain and culture are widely reported in many studies as barriers to MC [29,30,31,32,33,34], in addition to ethnicity, masculinity, sexuality, limited access, cost, pain, being attended to by female providers, lack of privacy, and secrecy [35]. A recent systematic review identified cultural influence, religion, fear, pain, and negative perceptions towards MMC as major barriers [36].

In this study, the practice of MMC was associated with older age, excellent knowledge, and awareness about the benefits of MMC. The association between the practice of MMC and older age is not surprising since majority of males get circumcised below the age of 20 years in traditionally circumcising communities [7]. Our finding on association of MMC with knowledge and awareness about the benefits of MMC is similar to a study that was conducted in Zambia [37]. The effect of knowledge and awareness on public health interventions is well known and highlights the importance of educational interventions and awareness campaigns to improve the uptake of MMC.

The majority (75%) of males in this study had good knowledge of the benefits of MMC, with a median knowledge of 62.5%. This finding is consistent with the findings from previous studies conducted in traditionally circumcising communities [38,39,40,41]. Younger men (32%), traditionally circumcised men (38%), and those with a negative attitude (34%) were more likely to have good knowledge about MMC. The level of knowledge was likely to increase with increased awareness. For instance, about 60% of those who had high awareness about the benefits of MMC were more likely to have excellent knowledge. This suggests that increasing awareness of the benefits of circumcision for HIV prevention is associated with a significant increase in knowledge about the benefits of MMC. On the other hand, excellent knowledge was associated with being aged ≥20 years (27%), a positive attitude towards MMC (27%), and being circumcised (22%). The further analysis revealed that having a positive attitude about MMC and being medically circumcised were the major determinants of excellent knowledge of MMC. The relationship between attitude, awareness, and knowledge is well-established in the scientific literature [41,42,43,44]. Furthermore, this relationship can be explained in terms of a dose-response [45,46]. More awareness campaigns are therefore needed to improve the knowledge of males about the benefits of MMC.

Although 42% of the study participants indicated that they would not be friends with medically circumcised men, almost 60% of men demonstrated a positive attitude towards MMC. These findings are consistent with the findings from a similar study conducted in 2005 in Namibia and Zimbabwe [38,47]. Similarly, in a study conducted by Mavhu in 2011, the majority of men (65%) had positive attitudes towards MC [48]. Older age, being from Ntabankulu, being Hlubi, being a Christian, and being medically circumcised were major determinants of a positive attitude towards MMC. Previous studies also found that ethnicity, religion, and circumcision status may influence the attitude of males towards MMC [29,30,31,32,33,34]. The association between a positive attitude towards MMC and Ntabankulu villages is unclear. However, we believe this may be explained by heightened awareness and lived experiences including adverse historical events seen over the years following the practice of TMC in Ntabankulu villages. In 2013, for instance, the Alfred Nzo District reported the highest number of illegal initiates and the highest number of hospital admissions due to injuries and deaths following TMC [49], with Ntabankulu villages being the worst affected. According to the parliamentary report in 2013, most reported injuries and deaths were attributed to assault, dehydration, infections, and mutilation during the initiation process [49]. The report also highlighted that “the custom of TMC had been hijacked by criminals who had total disregard for human life and only carried out the practices for commercial purposes, and some traditional surgeons performed the procedure under the influence of alcohol” [49]. All these factors may have played a significant role in the number of reported injuries and deaths associated with TMC. These historical events may have affected the attitudes of traditionally circumcising communities in Ntabankulu to favour MMC over TMC.

Most men believed that TMC proves manhood and strongly believed that this practice does not need to be changed or re-introduced. This is not surprising, considering that in traditionally circumcising communities, MC is believed to be a developmental milestone for a man [2,9,10]. TMC is a rite of passage for young adult males, with the main purpose of integrating them into adult society, building character, and shaping their personal discipline, livelihood skills, family life skills, community life, and village systems [2,9,10]. This explains why culture, instead of medical benefits, remains a major driver of circumcision in most circumcising communities [6,7,8].

More than half (52%) of the study participants believed that circumcised men have more sexual feelings and enjoy sex more than uncircumcised men. This is consistent with the findings from studies conducted in Jamaica [50] and Malawi [51]. However, uncircumcised men were less likely to believe this, and this finding is consistent with a study conducted in Botswana [52]. It is difficult to verify whether circumcised men have more sexual feelings and whether they enjoy sex more than uncircumcised men. However, in a sexual function and satisfaction survey conducted before and after circumcision among adult men participating in an RCT in Kenya, circumcised men reported increased ease of reaching orgasm, penile sensitivity, and more frequent sex compared to uncircumcised men [53].

Although most men realize the need to use a condom during sexual intercourse irrespective of circumcision status, some (30%) respondents from this study believed that they could have unprotected sex at any time because circumcision is 100% protective against HIV infection. This false sense of security and immunity against HIV infection was also reported in a study conducted in 2003 in South Africa [36] and could translate into risky sexual behavioural practices. However, 38% of respondents believed that circumcision reduces the chance of becoming infected with sexually transmitted infections.

Forty nine percent of uncircumcised men indicated that they would consider being medically circumcised. Although this level of acceptability is similar to the findings from the Botswana study, it is much lower than the median acceptability rate of 65% reported among uncircumcised men in Sub-Saharan African countries [29,48]. Sixty nine percent of male parents supported MMC for their sons. This was similar to the median acceptability rate of 71% reported among male parents from the systematic review conducted in 2007 in Sub-Saharan African countries [29]. Overall, 68% of our study participants accepted the involvement of health workers in MC, suggesting that MMC was generally accepted in Alfred Nzo although some participants believed that the practice of TMC does not need to be changed or re-introduced.

Older males were more likely to accept MMC compared to younger males. In contrast, a qualitative study conducted in Kenya found that being an older male was a barrier to VMMC because older men were not sexually active and were thus less likely to contract HIV [54]. The high acceptability of MMC among older males in this study may be influenced by context-specific factors and lived experiences rather than HIV prevention. For instance, adverse events such as death following the practice of TMC may have influenced older males to prefer MMC over TMC for safety reasons in their traditionally circumcising communities. Additionally, this study found that the acceptability of MMC was associated with excellent knowledge and awareness about the benefits of MMC, suggesting that these were major determinants of MMC.

The association of MMC acceptability with awareness is similar to previous studies [55]. However, the association with knowledge has not been consistent across studies. Some studies found that there was no link between knowledge, attitude, and acceptability of MMC [56,57], while others found that males with low knowledge were less likely to be medically circumcised [37,58]. Despite the inconsistencies, our results reaffirm the paradigm that awareness campaigns and educational interventions may likely influence the uptake of MMC. In addition, this study found that ethnicity, positive attitude, and being medically circumcised were major factors predicting the acceptability of MMC as an HIV biomedical intervention, similar to previous studies [37,59]. This highlights the need for a more people-centred approach through community dialogues, social mobilization, and regular health education.

### Study Limitations

This study may have been affected by recall bias commonly found in data from cross-sectional surveys. This may have affected the ability to recall some answers about knowledge and awareness about MMC and TMC among study participants, leading to underestimation or overestimation of the study findings. The study may be affected by reporting bias, leading to distortion of the measure of association (i.e., overestimation and/or underestimation) and study findings. Report biases are also common since questionnaires and surveys do not always result in accurate reporting with no ways of verifying the facts about obtained information.

Additionally, social desirability is likely to occur in settings where there is extensive publicity in favour of MMC. This may lead to overestimation of attitude, practices, and acceptability of MMC due to a desire to appear to hold contemporary views and approval of MMC of the study participants. The Stata regression analysis procedure automatically removes variables that are collinear. However, although there was no multi-collinearity detected, this may have affected the association between awareness, knowledge, and MMC acceptability, resulting in an underestimation or overestimation of the measure of association. Cross-sectional study design only provides a snapshot of a single moment in time and does not factor in other variables before or after this snapshot is taken. This may result in temporality bias. Furthermore, the results of the study may not be generalizable to other settings. Lastly, this study was conducted more than 5 years ago and a similar study in this subpopulation might report slightly different findings at this point in time.

## 5. Conclusions

In this study, we found that MC was a common practice in Alfred Nzo District. Although TMC was still preferred, traditional circumcising communities were slowly gravitating towards MMC, and a traditional setting was still preferred over a medical setting irrespective of circumcision type. Most of the study participants demonstrated a good knowledge of the benefits of MMC with many accepting the MMC program as a biomedical intervention for HIV reduction. In addition, knowledge, attitude, and awareness about the benefits of MMC played a significant role in the acceptance and practice of MMC, highlighting the importance of educational interventions and awareness campaigns to improve the uptake of an MMC programme in traditionally circumcising communities. Messaging on educational interventions and awareness campaigns could be tailored specifically to consider dynamics and differences in terms of ethnic subgroups, religion, and local context. Some circumcised men demonstrated a false sense of security and immunity against HIV infection, which may translate into risky sexual behavioural practices. Targeted educational interventions are needed to correct myths and false beliefs about MC in this population group. More robust awareness campaigns and people-centred approaches through community dialogues, social mobilization, and regular health education are needed to improve the level of knowledge and change the negative perceptions and attitude towards MMC. Serious complications and adverse events following TMC are common in Alfred Nzo District. Integration of TMC with MMC may significantly reduce complications associated with TMC in traditionally circumcising communities. To facilitate the integration between MMC and TMC, a special team of trained circumcised healthcare workers can be considered to conduct outreach programs during circumcision seasons in collaboration with the traditional authorities, traditional surgeons, and traditional nurses. In addition, the wound care and MC procedure can be conducted in traditional settings irrespective of circumcision type under normal circumstances. The local authorities are encouraged to explore innovative ways to make the healthcare system more inclusive and transform it to accommodate the immediate needs of its local people in a culturally sensitive and acceptable manner.

## Figures and Tables

**Table 1:** Demographic characteristics of male respondents, Alfred Nzo District, South Africa, 2015.

Variable (N = 343)	Frequency (n)	Percentage (%)
Age group		
<20 years	204	59
≥20 years	139	41
Marital status		
Single	279	81
Married	33	10
Cohabitating	24	7
Other	7	2
Tribe		
Sotho	26	8
Other	17	5
Bhaca	26	8
Hlubi	26	8
Mpondo	101	29
Xhosa	147	43
Religion		
Christian	185	54
Traditional	140	41
Other	18	5
School attendance		
No	4	1
Yes	339	99
Level of education		
Primary	25	7
Secondary	302	88
Tertiary	16	5
Employment status		
Employed	44	13
Unemployed	61	18
Student	238	69
Rural town		
Maluti	33	10
Mount Ayliff	50	15
Mount Frere	53	15
Ntabankulu	65	19
Mbizana	142	41

**Table 2:** Level of knowledge about medical male circumcision among males, Alfred Nzo District, South Africa, 2015.

Variable (N = 343)	N	Poor (%)	Fair (%)	Good (%)	Excellent (%)	*p*-Value
Age in years						0.021
<20 years	204	40 (20%)	67 (33%)	65 (32%)	32 (16%)	
≥20 years	139	14 (10%)	45 (32%)	43 (42%)	37 (27%)	
Religion						0.009
Traditionalist	140	30 (21%)	45 (32%)	50 (36%)	15 (11%)	
Christian	185	22 (12%)	61 (33%)	52 (28%)	50 (27%)	
Other	18	2 (11%)	6 (33%)	6 (33%)	4 (22%)	
Employment status						0.037
Employed	44	3 (7%)	17 (39%)	13 (30%)	11 (25%)	
Unemployed	61	7 (11%)	19 (31%)	15 (25%)	20 (33%)	
Student	238	44 (18%)	76 (32%)	80 (34%)	38 (16%)	
Rural town						0.001
Maluti	33	2 (6%)	10 (30%)	12 (36%)	9 (27%)	
Mount Ayliff	50	9 (18%)	14 (28%)	16 (32%)	11 (22%)	
Mount Frere	53	7 (13%)	18 (34%)	17 (32%)	11 (21%)	
Ntabankulu	65	11 (17%)	17 (26%)	11 (17%)	26 (40%)	
Mbizana	142	25 (18%)	53 (37%)	52 (37%)	12 (8%)	
Circumcision type						<0.001
Traditionally circumcised	204	24 (12%)	71 (35%)	77 (38%)	32 (16%)	
Medically circumcised	55	4 (7%)	12 (22%)	12 (22%)	27 (49%)	
Uncircumcised	84	19 (23%)	28 (34%)	27 (31%)	10 (12%)	
Attitude towards MMC						<0.001
Negative	142	13 (22%)	49 (35%)	48 (34%)	14 (10%)	
Positive	201	23 (11%)	63 (31%)	60 (30%)	55 (27%)	

MMC, medical male circumcision.

**Table 3:** Factors associated with positive attitude towards medical male circumcision among males, Alfred Nzo District, South Africa, 2015.

Variable (N = 201)	Positive Attitude		Bivariate Analysis			Multivariate Analysis		
	N	%	Crude OR	95% CI	*p*-Value	Adjusted OR	95% CI	*p*-Value
Age in years								
<20 years	104	51	1	Reference	Reference	1	Reference	Reference
≥20 years	97	70	2.22	1.41–3.50	0.001	2.65	1.29–5.45	0.008
Tribe								
Sotho	10	38	1	Reference	Reference	1	Reference	Reference
Other	8	47	1.4	0.41–4.90	0.577	1.1	0.15–8.28	0.928
Bhaca	17	65	3.02	0.98–9.36	0.055	5.18	0.87–30.91	0.071
Hlubi	19	73	4.34	1.34–14.03	0.014	5.15	1.02–25.94	0.047
Mpondo	64	63	2.77	1.14–6.72	0.025	4.05	1.06–15.47	0.041
Xhosa	83	56	2.08	0.88–4.88	0.094	3.58	1.04–12.37	0.044
Religion								
Traditionalist	67	48	1	Reference	Reference	1	Reference	Reference
Other	7	39	0.29	0.11–0.79	0.015	0.47	0.23–0.82	0.008
Christian	127	69	2.39	1.51–3.76	<0.001	2.65	1.40–5.03	0.003
Rural town								
Maluti	16	48	1	Reference	Reference	1	Reference	Reference
Mount Ayliff	35	70	2.48	1.00–6.17	0.051	3.07	0.89–10.64	0.077
Mount Frere	26	49	1.02	0.43–2.44	0.959	0.81	0.23–2.84	0.741
Ntabankulu	50	77	3.54	1.45–8.66	0.006	4.36	1.25–15.18	0.021
Mbizana	74	52	0.94	0.48–1.86	0.707	1.33	0.43–4.11	0.623
Circumcision status								
Circumcised	148	56	1	Reference	Reference	1	Reference	Reference
Uncircumcised	53	68	2.12	1.32–3.41	0.002	2.65	1.40–5.03	0.003
Circumcision type *								
Traditionally circumcised	96	47	1	Reference	Reference	1	Reference	Reference
Medically circumcised	52	95	19.5	5.90–64.47	<0.001	16.08	4.19–61.75	<0.001

OR, odds ratio; CI, confidence interval; * 53 uncircumcised men were excluded in the sub-analysis. The following variables were included in the multivariable model: age in years, tribe, religion, name of the rural town, circumcision status, and circumcision type.

**Table 4:** Factors associated with being medically circumcised among males, Alfred Nzo District, South Africa, 2015.

Variable (N = 55)	MMC		Bivariate Analysis			Multivariate Analysis		
	N	%	Crude OR	95% CI	*p*-Value	Adjusted OR	95% CI	*p*-Value
Age in years								
<20 years	16	12	1	Reference	Reference	1	Reference	Reference
≥20 years	39	30	3.03	1.59–5.76	0.001	2.44	1.23–4.84	0.011
Employment status								
Unemployed	16	28	1	Reference	Reference			
Employed	17	40	1.74	0.75–4.06	0.198			
Student	22	14	0.41	0.20–0.85	0.017			
Rural town								
Maluti	4	14	1	Reference	Reference			
Mount Ayliff	8	21	1.67	0.45–6.20	0.446			
Mount Frere	7	16	1.22	0.23–4.60	0.774			
Ntabankulu	28	50	6.25	1.92–20.31	0.002			
Mbizana	8	9	0.59	0.16–2.12	0.417			
Attitude towards MMC								
Negative	3	3	1	Reference	Reference			
Positive	52	35	19.5	5.90–64.47	<0.001			
Awareness								
No	4	7	1	Reference	Reference	1	Reference	Reference
Yes	51	26	4.94	1.71–14.31	0.003	3.26	1.08–9.86	0.037
Knowledge								
Poor	4	14	1	Reference	Reference	1	Reference	Reference
Fair	12	14	1.01	0.30–3.44	0.982	0.89	0.25–3.16	0.863
Good	12	13	0.94	0.28–3.17	0.914	0.72	0.20–2.53	0.605
Excellent	27	46	5.06	1.56–16.41	0.007	3.38	1.00–11.49	0.05

MMC, medical male circumcision; OR, odds ratio; CI, confidence interval. The following variables were included in the multivariable model: age in years, awareness, and knowledge.

**Table 5:** Factors associated with acceptability of medical male circumcision among males, Alfred Nzo District, South Africa, 2015.

Variable (N = 196)	Accept MMC		Bivariate Analysis			Multivariate Analysis		
	N	%	Crude OR	95% CI	*p*-Value	Adjusted OR	95% CI	*p*-Value
Age in years								
<20 years	105	51	1	Reference	Reference			
≥20 years	91	65	1.79	1.15–2.79	0.01			
Tribe								
Sotho	7	27	1	Reference	Reference	1	Reference	Reference
Other	10	59	3.88	1.06–14.19	0.041	9.26	1.56–55.13	1.14
Bhaca	16	62	4.34	1.34–14.03	0.014	2.95	0.68–12.78	0.149
Hlubi	13	50	2.71	0.85–8.64	0.091	1.67	0.40–6.98	0.483
Mpondo	57	56	3.52	1.36–9.11	0.01	3.54	1.04–12.09	0.043
Xhosa	93	63	4.67	1.85–11.84	0.001	5.05	1.53–16.66	0.008
Employment status								
Employed	31	70	1	Reference	Reference			
Unemployed	47	77	3.36	1.85–6.10	<0.001			
Student	118	50	0.29	0.15–0.56	<0.001			
Rural town								
Maluti	18	55	1	Reference	Reference			
Mount Ayliff	28	56	1.06	0.44–2.57	0.896			
Mount Frere	29	55	1	0.42–2.41	0.988			
Ntabankulu	50	77	2.78	1.13–6.80	0.025			
Mbizana	71	50	0.83	0.39–1.78	0.638			
Attitude towards MMC								
Negative	52	37	1	Reference	Reference	1	Reference	Reference
Positive	144	72	4.37	2.76–6.92	<0.001	2.35	1.30–4.35	0.005
Awareness								
No	37	43	1	Reference	Reference			
Yes	159	62	2.22	1.35–3.63	0.002			
Knowledge								
Poor	24	44	1	Reference	Reference	1	Reference	Reference
Fair	60	54	1.44	0.75–2.77	0.271	1.32	0.42–3.01	0.822
Good	59	55	1.51	0.78–2.90	0.223	1.1	0.42–2.90	0.845
Excellent	53	77	4.14	1.91–8.99	<0.001	3.07	0.99–9.58	0.053
Circumcision type *								
TMC	105	51	1	Reference	Reference			
MMC	46	84	4.82	2.24–10.36	<0.001			

MMC, medical male circumcision; TMC, traditional male circumcision; OR, odds ratio; CI, confidence interval; * 45 uncircumcised men were excluded in the sub-analysis. The following variables were included in the multivariable model: tribe, attitude towards MMC, and knowledge towards MMC.

## Data Availability

All the data supporting our findings are contained within this manuscript. Datasets used in this study may be requested from the main author: Thobani Ntshiqa (email: thobanintshiqa@yahoo.com).

## Appendix A

**Table A1:** Circumcision status, attitude, circumcision type, and acceptability of medical male circumcision by demographic characteristics, Alfred Nzo District, South Africa, 2015.

Variable (N = 343)	Circumcision Status					Attitude towards MMC					Circumcision Type					Accept MMC				
	Circumcised		Uncircumcised		*p*-Value	Negative		Positive		*p*-Value	TMC		MMC		*p*-Value	No		Yes		*p*-Value
	n	%	n	%		n	%	n	%		n	%	n	%		n	%	n	%	
Age in years					<0.001					0.001					0.001					0.01
<20 years	132	50	72	92		100	70	104	52		113	55	16	29		99	67	105	54	
≥20 years	133	50	6	8		42	30	97	48		91	45	39	71		48	33	91	46	
Tribe					0.397					0.1					0.015					0.026
Sotho	21	8	5	6		16	11	10	5		16	8	3	5		19	13	7	4	
Other	13	5	4	5		9	6	8	4		8	4	4	7		7	5	10	5	
Bhaca	21	8	5	6		9	6	17	8		12	6	9	16		10	7	16	8	
Hlubi	24	9	2	3		7	4	19	9		19	9	5	9		13	9	13	7	
Mpondo	73	28	28	36		37	26	64	32		52	25	20	36		44	30	57	29	
Xhosa	113	43	34	44		64	45	83	41		97	48	14	25		54	37	93	47	
Rural town					0.001					0.002					<0.001					0.009
Maluti	30	11	3	4		17	12	16	8		25	25	4	7		15	10	18	9	
Mount Ayliff	38	14	12	15		15	11	35	17		30	30	8	15		22	15	28	14	
Mount Frere	45	17	8	10		27	19	26	13		36	36	7	13		24	16	29	15	
Ntabankulu	57	22	8	10		15	11	50	25		28	28	28	51		15	10	50	26	
Mbizana	95	36	47	60		68	58	74	32		85	85	8	15		71	48	71	36	
Awareness					0.341					0.001					0.001					0.001
No	64	24	23	29		49	35	38	19		57	28	4	7		50	34	37	19	
Yes	201	76	55	71		93	65	163	81		147	72	51	93		97	66	159	81	
Knowledge					<0.001					<0.001					<0.001					0.002
Poor	29	11	25	32		31	22	23	11		24	12	4	7		30	20	24	12	
Fair	87	33	25	32		49	35	63	31		71	35	12	22		52	35	60	31	
Good	90	34	18	23		48	34	60	30		77	38	12	22		49	33	59	30	
Excellent	59	22	10	13		14	10	55	27		32	16	27	49		16	11	53	27	

MMC, medical male circumcision; TMC, traditional male circumcision; P, *p*-value.
